# Efficacy of eHealth Technologies on Medication Adherence in Patients With Acute Coronary Syndrome: Systematic Review and Meta-Analysis

**DOI:** 10.2196/52697

**Published:** 2023-12-19

**Authors:** Akshaya Srikanth Bhagavathula, Wafa Ali Aldhaleei, Tesfay Mehari Atey, Solomon Assefa, Wubshet Tesfaye

**Affiliations:** 1 Department of Public Health College of Health and Human Services North Dakota State University Fargo, ND United States; 2 Gastroenterology and Hepatology Department Mayo Clinic Jacksonville, FL United States; 3 Clinical Pharmacy Unit School of Pharmacy, College of Health Sciences Mekelle University Mekelle Ethiopia; 4 Department of Pharmacology and Clinical Pharmacy Addis Ababa University Addis Ababa Ethiopia; 5 Sydney Pharmacy School The University of Sydney NSW Australia

**Keywords:** medication adherence, eHealth, secondary prevention, acute coronary syndrome, heart disease, text messaging, mobile app, cardiology, cardioprotective, prevention, efficacy, statins

## Abstract

**Background:**

Suboptimal adherence to cardiac pharmacotherapy, recommended by the guidelines after acute coronary syndrome (ACS) has been recognized and is associated with adverse outcomes. Several randomized controlled trials (RCTs) have shown that eHealth technologies are useful in reducing cardiovascular risk factors. However, little is known about the effect of eHealth interventions on medication adherence in patients following ACS.

**Objective:**

The aim of this study is to examine the efficacy of the eHealth interventions on medication adherence to selected 5 cardioprotective medication classes in patients with ACS.

**Methods:**

A systematic literature search of PubMed, Embase, Scopus, and Web of Science was conducted between May and October 2022, with an update in October 2023 to identify RCTs that evaluated the effectiveness of eHealth technologies, including texting, smartphone apps, or web-based apps, to improve medication adherence in patients after ACS. The risk of bias was evaluated using the modified Cochrane risk-of-bias tool for RCTs. A pooled meta-analysis was performed using a fixed-effect Mantel-Haenszel model and assessed the medication adherence to the medications of statins, aspirin, P2Y12 inhibitors, angiotensin-converting enzyme inhibitors or angiotensin receptor blockers, and β-blockers.

**Results:**

We identified 5 RCTs, applicable to 4100 participants (2093 intervention vs 2007 control), for inclusion in the meta-analysis. In patients who recently had an ACS, compared to the control group, the use of eHealth intervention was not associated with improved adherence to statins at different time points (risk difference [RD] –0.01, 95% CI –0.03 to 0.03 at 6 months and RD –0.02, 95% CI –0.05 to 0.02 at 12 months), P2Y12 inhibitors (RD –0.01, 95% CI –0.04 to 0.02 and RD –0.01, 95% CI –0.03 to 0.02), aspirin (RD 0.00, 95% CI –0.06 to 0.07 and RD –0.00, 95% CI –0.07 to 0.06), angiotensin-converting enzyme inhibitors or angiotensin receptor blockers (RD –0.01, 95% CI –0.04 to 0.02 and RD 0.01, 95% CI –0.04 to 0.05), and β-blockers (RD 0.00, 95% CI –0.03 to 0.03 and RD –0.01, 95% CI –0.05 to 0.03). The intervention was also not associated with improved adherence irrespective of the adherence assessment method used (self-report or objective).

**Conclusions:**

This review identified limited evidence on the effectiveness of eHealth interventions on adherence to guideline-recommended medications after ACS. While the pooled analyses suggested a lack of effectiveness of such interventions on adherence improvement, further studies are warranted to better understand the role of different eHealth approaches in the post-ACS context.

## Introduction

Acute coronary syndrome (ACS) occurs due to the blockage of 1 or more coronary arteries, which often leads to chest pain, myocardial infarction, and other serious complications. It has a high recurrence rate among individuals who previously had ACS [1,2], necessitating the need for a range of pharmacotherapeutic interventions during and post incident [3]. As such, people with ACS typically require multiple medications including aspirin, β-blockers, and statins to prevent future cardiac events [4]. Current guidelines recommend the long-term use of 5 classes of medications in secondary prevention following ACS: aspirin, statins, β-blockers, angiotensin-converting enzyme inhibitors (ACEIs) or angiotensin receptor blockers (ARBs), and in addition P2Y12 inhibitors for 1 year to reduce future ACS incidents and associated cardiac complications. Further, people with ACS may also require medications to manage symptoms like chest pain or to prevent disease complications including blood clots and myocardial infarction, resulting in an overall increase in medication burden.

Adherence to medications is defined as “The process by which patients take their medications as prescribed, composed on initiation, implementation and discontinuation [5].” While there remains a lack of consensus on what is considered an adequate level of medication adherence [6], evidence indicates that suboptimal adherence to chronic medications is a widely recognized clinical challenge that places a significant burden on health care expenditure [7]. Reports showed that medication nonadherence is a highly prevalent clinical problem, which varies based on the disease condition, age, study setting, and definition of medication adherence [8]. Evidence from a systematic review and meta-analysis shows that adherence to secondary prevention pharmacotherapy ranges between 54% and 86% within 1 year of discharge from the hospital for ACS [9], with no consistent predictors of nonadherence identified across all cardiac medication classes. This is further supported by another meta-analysis that also reported poor adherence to secondary prevention medications in people with coronary heart disease, with little differences among medication classes [10].

While poor medication adherence could be a conscious decision in certain circumstances, unintentional nonadherence, for example, due to cognitive and memory issues, plays a significant role in predicting poor medication adherence [11]. Factors contributing to unintentional nonadherence are considered amenable to changes through appropriate interventions. eHealth-based interventions are emerging as an integral component of health care service delivery and are contributing to improved health outcomes. Web-based eHealth technologies like SMS text messages or interactive voice response, mobile apps, and calls as modes of providing adherence telefeedback have been successfully tested on a range of medical conditions, leading to improved adherence to long-term medications [12].

Emerging evidence from studies on the efficacy of eHealth interventions in improving medication adherence in people with ACS has led to inconclusive findings [13-17]. Therefore, in this study, we aim to (1) conduct a systematic review and meta-analysis to investigate the effectiveness of eHealth interventions in improving medication adherence in people with recent history of ACS and (2) examine any subgroup differences in the effectiveness of such interventions based on their application on different medication classes prescribed to manage ACS or method of adherence assessment.

## Methods

### Study Design

The study involves a systematic review and meta-analysis of randomized controlled trials (RCTs) and was designed in accordance with the PRISMA (Preferred Reporting Items for Systematic Reviews and Meta-Analysis) statements (Multimedia Appendix 1) [18].

### Data Search Strategy

Electronic data searches of PubMed, Embase, Scopus, and Web of Science were conducted between May and October 2022, with an update in October 2023 to identify RCTs focusing on eHealth interventions to improve pharmacotherapy adherence in patients who had an ACS incident. Articles published from January 2000 to October 2023 were considered for screening. The following search keywords or concepts and relevant synonyms words were used combined via appropriate Boolean operators: Mobile health, text messages, smartphones, eHealth, and mobile applications in combination with cardiovascular disease, secondary prevention, adherence, medication adherence, medication nonadherence, coronary artery disease, acute coronary syndrome, myocardial infarction, and cardiac rehabilitation. The detailed keywords and search strategies used in different databases are presented in Multimedia Appendix 2.

The Population Intervention Comparison Outcome(s) Study design statement for this systematic review was as follows: participants: patients with post-ACS; intervention: eHealth technology (telehealth, eHealth, smartphone, texting, mobile health [mHealth], phone apps, etc); comparisons: standard or usual care; outcomes: adherence to guidelines-recommended post-ACS pharmacotherapy; and study design: RCTs.

### Selection Criteria

For this systematic review, studies were included if they investigated the effectiveness of eHealth technologies (eg, mobile phone app, a web-based app, a smartphone app, an electronic device, or texting) in an RCT for a duration of at least 12 weeks. The studies were focused on improving adherence to various classes of guideline-recommended cardioprotective medications, such as aspirin, statins, β-blockers, P2Y12 inhibitors, and ACEIs or ARBs, among patients after ACS. The studies, to be included in the meta-analysis, had to provide information on the number of subjects or proportion of use for the 5 classes of medications indicated for secondary prevention after ACS in both the intervention and control groups at baseline as well as at follow-up periods. Studies that examined eHealth interventions for the treatment of obesity, hypertension, dyslipidemia, secondary lifestyle factors, and smoking cessation in patients without ACS were excluded. Articles published in non-English language results were also excluded. Additionally, abstracts, case reports, editorials, and conference presentations were not considered for this systematic review.

### Screening Process

Titles and abstracts found through the electronic database searches were imported into the Covidence systematic review software (Veritas Health Innovation Ltd). A total of 2 reviewers (ASB and WT) independently screened the abstracts. Studies not meeting the predetermined selection criteria were excluded. After reviewing the abstracts of identified studies, the full texts of eligible publications were subsequently evaluated by the same 2 independent reviewers for potential inclusion in the final analysis. In addition, bibliographies of relevant publications were also examined to identify any articles missed during the original database searches. Any disagreements regarding article inclusion and exclusion of the article were resolved through collaborative review. The final results were reviewed by all authors.

### Data Extraction and Critical Appraisal

A total of 2 authors (ASB and WAA) independently extracted the data into a predetermined Excel (Microsoft Corp) spreadsheet, and the third author (WT) evaluated the collected data. Study characteristics (authors, year of publication, country, registration, design, and duration of the trial protocol); patient demographics (mean age, sex, size, and type of control condition); design parameters (type of intervention and length of intervention); eHealth intervention features (type of electronic device, messaging frequency, and a web-based app), and outcomes (method and frequency of assessment for adherence and type of medication) were extracted.

### Risk of Bias of Individual Studies

A total of 2 independent authors evaluated the risk of bias within individual studies using the modified Cochrane risk-of-bias tool for RCTs [19], a 7-item instrument that assesses selection bias, allocation concealment, implementation bias, measurement bias, follow-up bias, reporting bias, and others. Any discrepancies were resolved by consensus.

### Outcomes

The primary outcome includes overall adherence to cardioprotective medication classes (statins, aspirin, P2Y12 inhibitors, ACEI or ARBs, and β-blockers) in patients with ACS following eHealth interventions. The secondary goal is to evaluate the effectiveness of eHealth interventions on medication adherence at 6-month and 12-month follow-up periods.

### Data Analysis

The included trials reported the extent of medication nonadherence, including to specific drug classes, using both self-report questionnaires and objective methods like medication possession ratio or prescription claims data at different time intervals (3-, 6- or 12-months post intervention). When trials reported multiple follow-up assessments, we pooled the relevant data to determine the effect of the intervention at 6-month and 12-month time points. To analyze the data, effect sizes were estimated using standardized risk differences (RD) between the intervention and control groups, as well as RD for each cardiovascular medicine in the study and weighted pool estimates. We considered variations in outcome measurement across studies by applying appropriate statistical methods (ie, fixed effects for heterogeneity level of ≤25% and random effects when heterogeneity level was >25%) to generate meta-analytic estimates of intervention effect and presented as RD [19]. A chi-square test was used to assess homogeneity between studies and a homogeneity *P* value of less than .10 was considered statistically significant [20]. Subgroup analyses were performed using self-report and objective adherence assessment methods to determine the effectiveness of intervention on adherence to individual or all drug classes both at 6-month and 12-month follow-ups. Due to the small number of studies meeting our inclusion criteria, publication bias was not assessed [21]. All statistical analyses were performed using STATA MP statistical software (version 16.1; StataCorp). The results were expressed as an “RD” with 95% CI. A *P* value less than .05 was considered a statistically significant difference between the intervention and the controls.

## Results

### Overview

**Figure 1:**
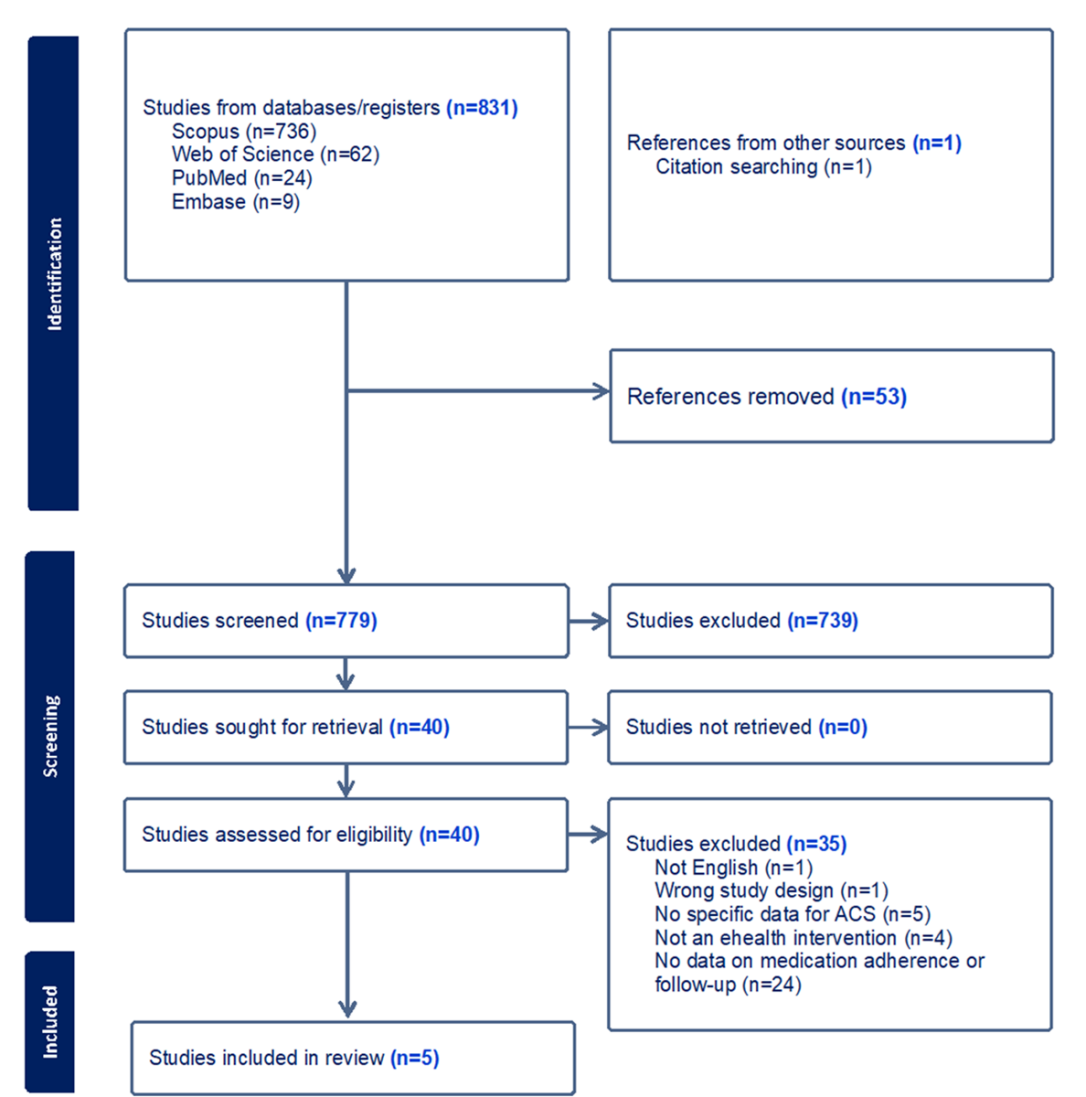
Covidence PRISMA flow diagram showing article screening and selection criteria. ACS: acute coronary syndrome; PRISMA: Preferred Reporting Items for Systematic Reviews and Meta-Analysis.

The database search resulted in 831 titles and abstracts, while the bibliographic search of these articles did not result in any other articles. Of the 831 articles screened, 779 articles were considered for screening and 40 articles met the eligibility criteria, but most of these RCTs did not have data on medication adherence or follow-up (n=24), no specific data on ACS (n=5), were not an eHealth intervention (n=4), and other reasons (n=4). Finally, 5 peer-reviewed journal articles met the inclusion criteria (Figure 1) [13-17] and their features are summarized in Table 1.

All RCTs were published in English between the years 2020 and 2022. A total of 3 of the included studies were from China, while 1 was conducted in Australia and another 1 in New Zealand. The 5 studies included 4100 participants, with 2093 and 2007 people included in the intervention and control groups, respectively. The majority of the targeted participants were male, with their average age ranging from 57 to 64.8 years.

Adherence to statins, aspirin, P2Y12 inhibitors, ACEIs or ARBs, and β-blockers was evaluated using text message, WeChat, and smart phone–based app interventions. Both self-report [13-15,17] and objective [13,15] adherence measurement methods were used by the included studies to determine the level of nonadherence to prescribed medications. For example, 2 studies used the 8-item Morisky medication adherence scale (MMAS-8) to determine the proportion of medication nonadherence [15,17], while other studies used self-reports along with objective methods like prescription claims data [13], or a medication possession ratio [15]. Patients were deemed adherent, for example, if at different time points, they had a medication possession ratio of ≥80% (determined based on the number of days patients are in possession of the dispensed drugs divided by the number of days used for follow-up) [15] or self-report of medication use >80% of the time in the previous 30-days (24/30 days) [13]. People were also considered adherent based on having a score of ≥6 using the MMAS-8 scale, which was obtained mainly from “yes” and “no” responses and 1 Likert-based question [17].

**Table 1:** Characteristics of included studies in the meta-analysis.

Study	Year	Country	Participants, n		Mean age, mean (SD)	Male, %	Randomization, n:n	eHealth intervention type and frequency	Medications	Assessment method	Adherence cut-off, %	Duration of intervention, months
			Intervention	Control								
Chow et al [13]	2022	Australia	716	708	58.0 (10.7)	79.2	1:1	A 1-year program of customized SMS text messages (4 per week in the first 6 months, followed by 3 per week in the subsequent 6 months)	Statins, other antiplatelet^a^, aspirin, ACEI^b^ or ARB^c^, and β-blockers	Prescription claims data (PBS^d^). Self-report on medication use over past 30 days	>80	6 and 12
Wang et al [14]	2022	China	81	83	62 (12.4)	82.8	1:1	Provision of information related to medications and lifestyle factors coupled with a 12-month WeChat app-based follow-up	Statins, aspirin, and ACEI or ARB	Interviewer-led questionnaire	>80	6 and 12
Maddison et al [15]	2021	New Zealand	153	153	61 (11)	77.1	1:1	Personalized, automated self-management program delivered via SMS text messages (1 message per day for 24 weeks plus 35 additional messages in the first 12 weeks)	Statins, aspirin, ACEI or ARB, and β-blockers	MPR^e^ and MMAS-8^f^	80	6 and 12
Shi et al [16]	2021	China	642	564	64.8 (10.6)	72.6	1:1	Provision of health lifestyle recommendations and medication advice with a WeChat-based telemedicine management app for follow-up	Statins, antiplatelet, ACEI or ARB, and β-blockers	Self-report	90	1, 3, 6, and 12
Yu et al [17]	2020	China	501	499	57.3 (9.0)	85.5	1:1	Provision of information using a smartphone app (Heart Health) on medications, including reminders post discharge	Statins, antiplatelet, ACEI or ARB, and β-blockers	MMAS-8	N/A^g^	3 and 6

^a^P2Y12 antagonists or ticagrelor.

^b^ACEI: angiotensin-converting enzyme inhibitors.

^c^ARB: angiotensin receptor blocker.

^d^PBS: Pharmaceutical benefits scheme.

^e^MPR: medication possession ratio.

^f^MMAS-8: 8-item Morisky Medication Adherence Scale.

^g^N/A: not applicable.

### The Effectiveness of eHealth Intervention on Adherence to Cardioprotective Medications

**Figure 2:**
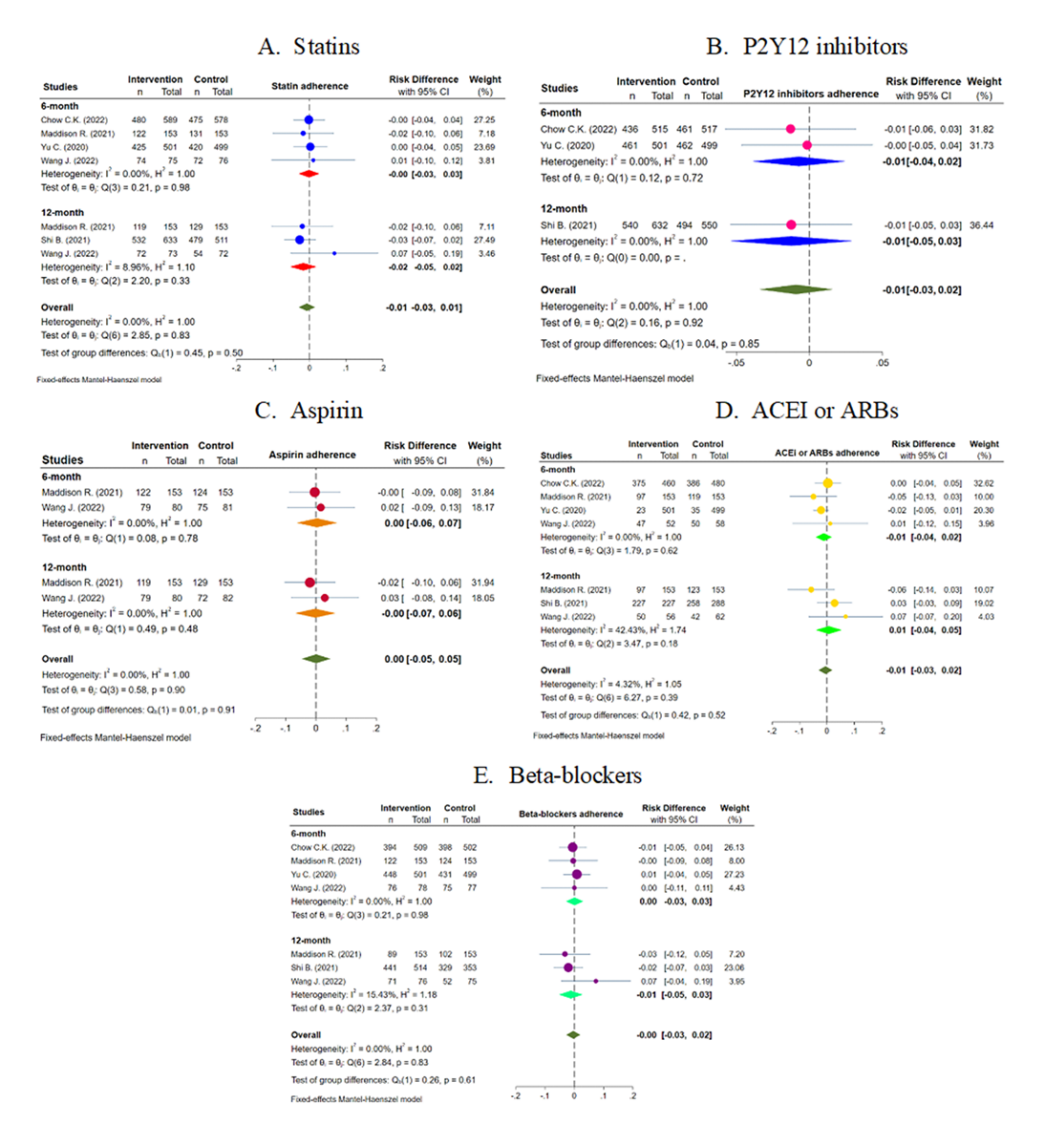
Effect of eHealth intervention on medication adherence after acute coronary syndrome. ACEI: angiotensin-converting enzyme inhibitor; ARB: angiotensin receptor blocker [13-17]. For a higher-resolution version of this figure, see Multimedia Appendix 3.

Figure 2 shows the weighted RD between the intervention and the control groups for statins, P2Y12 inhibitors, ACEIs or ARBs, β-blockers, and aspirin. A total of 4 studies provided data for intervention on adherence to statins, showing that eHealth intervention was not associated with significant improvement in adherence both at 6 months (RD –0.00, 95% CI –0.03 to 0.03; *I*^2^=0.0%) and 12 months (RD –0.02, 95% CI –0.05 to 0.02) post intervention. Similarly, no improvements in the medication adherence following eHealth intervention were observed for other classes of medications, such as P2Y12 inhibitors (RD –0.01, 95% CI –0.04 to 0.02; *I*^2^=0.0% at 6 months and RD –0.01, 95% CI –0.05 to 0.03; *I*^2^=0.0% at 12 months), aspirin (RD 0.00, 95% CI –0.06 to 0.07; *I*^2^=0.0% at 6 months and RD 0.00, 95% CI –0.07 to 0.06; *I*^2^=0.0% at 12 months), ACEIs or ARBs (RD –0.01, 95% CI –0.04 to 0.02; *I*^2^=0.0% at 6 months and RD 0.01, 95% CI –0.04 to 0.05; *P*=.18; *I*^2^=42.4% at 12 months), and β-blockers (RD 0.00, 95% CI –0.03 to 0.03; *I*^2^=0.0% at 6 months and RD –0.01, 95% CI –0.05 to 0.03; *P*=.31; *I*^2^=15.4% at 12 months).

### Subgroup Analyses for the Effect of Interventions Based on Adherence Assessment Method

A subgroup analysis of the effectiveness of eHealth interventions on medication adherence at 6 months’ and 12 months’ time points post intervention was conducted based on the adherence assessment methods used (self-report vs objective). The findings showed no significant combined RDs in overall medication adherence between intervention and control groups both at 6-month and 12-month follow-up periods, irrespective of the type of medication adherence assessment method applied (see detailed analyses in Multimedia Appendix 4). Heterogeneity among studies was high for most analyses, except when using the objective adherence assessment method. Furthermore, the studies that provided data for pooled estimation of the effect of the intervention on adherence to all drug classes also revealed no improvement in adherence both at 6 months (RD 0.01, 95% CI –0.00 to 0.02; *I*^2^=92.3%) and 12 months (RD 0.00, 95% CI –0.01 to 0.01; *I*^2^=1.00%).

### Quality Assessment of Included Studies

Overall, all studies showed a low risk of bias, and only 1 study did not provide a description of the blinding of outcome assessment. In Table 2, we reported the methodological quality assessment of individual studies as per the modified Cochrane risk of bias tool for RCTs.

**Table 2:** Risk of bias in included studies^a^.

Study	Selection bias		Performance bias	Detection bias	Attrition bias	Proportion bias	Outcome bias	Reporting bias	Treatment bias
	Random sequence generation	Allocation concealment	Blinding of participants and personnel	Blinding of outcome assessment	Incomplete outcome data	Groups balanced at baseline	Group received same intervention	Selective reporting	Intention-to-treat analysis
Chow et al [13]	Low	Low	Low	Low	Low	Low	Low	Low	Low
Wang et al [14]	Low	Low	Low	Low	Unclear	Low	Low	Low	Low
Maddison et al [15]	Low	Low	Low	Low	Unclear	Low	Low	Low	Low
Shi et al [16]	Unclear	Low	Unclear	Unclear	Low	Low	Low	Low	Unclear
Yu et al [17]	Low	Low	Low	High	Low	Low	Low	Low	Low

^a^Risk of bias: review authors’ judgments about each risk of bias item for the included studies.

## Discussion

### Principal Findings

This work presents a systematic review and quantitative analysis of randomized trials evaluating the effect of eHealth interventions on adherence to medications acting on the cardiovascular system in people who experienced ACS. The findings of this review revealed that eHealth-based intervention was not associated with significant improvements in adherence to guideline-recommended medications such as statins, P2Y12 inhibitors, aspirin, or hypertension medications or overall medication classes after ACS incidents. At 6 and 12 months’ time points after the intervention, no significant improvements in cardioprotective medication adherence were observed, irrespective of the methods of adherence assessment used.

Previous evidence from a meta-analysis of 16 randomized controlled trials found that sending medication-related reminders in the form of SMS doubled medication adherence across a range of chronic conditions [22]. This is in contrast to the findings of our review, which focused on patients with an acute medical condition on top of preexisting chronic diseases. ACS is a life-threatening condition that may require a specialized intervention plan. Consistent postdischarge assistance and education for long-term secondary prevention of ACS remain a health care service challenge. eHealth-based interventions are hypothesized to have the potential to improve the implementation and accessibility of nonpharmacological modalities for promoting the long-term secondary prevention of ACS [23]. These low-cost, easy-to-implement, and scalable eHealth interventions may have used as a consolidated source of information. Neither do they require extra investment or extensive clinician involvement.

Despite being low-cost and scalable, however, the contents of eHealth messages may not be enough to elicit behavioral changes in patients with ACS. This is particularly difficult in contexts where there are competing health goal priorities affecting medication adherence, like in case of patients experiencing acute illnesses superimposed on chronic conditions.. Similar initiatives that were published in the past, that addressed a single behavior, for example, such as exercise or smoking cessation, reported the success of eHealth intervention [24,25]. Targeting multiple behaviors simultaneously may be burdensome for patients demanding both acute and chronic medical care. SMS text messaging program alone may not satisfy participants’ needs for more personalized interventions [26].

Prior research demonstrates that eHealth technologies can address extrinsic social and economic factors to improve medication adherence after ACS [27-29]. For example, the study by Redfern et al [27] highlighted how an iterative, theory-based development process for texting interventions can inform optimized eHealth tools to enhance post-ACS pharmacotherapy adherence. Their user-centered approach integrating behavior change techniques suggests that future eHealth interventions could benefit from participatory design tailored to patient needs and preferences.

Applying such standardized methodology grounded in guidelines and tailored content may advance the effectiveness of eHealth adherence promotion after ACS. In addition, another pilot study by Riegel et al [29] tested a personalized telehealth intervention using behavioral economics and financial incentives to promote maintained aspirin adherence after ACS hospitalization, representing a participatory approach tailored to patient motivations that showed promising trends versus declining adherence in the controls.

From our pooled analysis, the findings revealed that the implemented eHealth programs were not significantly more effective than usual care in improving cardioprotective medication adherence in people who experienced recent ACS, regardless of the adherence assessment method used. This may point to the need for a redesign of eHealth interventions’ contents or components as well as their delivery. Based on the literature, for interventions to be more effective, the design should be theory-driven, iteratively designed, and culturally tailored to provide educational and motivational information to patients who experienced ACS. A successful intervention outcome may result from incorporating personalized intervention during a design process [29]. Most of the studies included in our analyses did not use theory-driven interventional materials, which are found to be effective in improving self-management practices and adherence in certain health conditions and patient populations [30-32]. The application of theory-based interventions provided in the right context may lead to improved adherence to treatments. However, it is important to note that the given patients, who were included in the targeted studies, were enrolled at or soon after their hospital discharge, they are more likely to have higher adherence and lifestyle adjustments, regardless of the implementation of an intervention.

This review synthesized evidence from published studies of relatively high quality, with all included studies being RCTs. Due to the rigor of their study designs, the majority of articles included in the meta-analysis had a minimal risk of bias. However, our evidence from the systematic review should be interpreted in the context of several major limitations. Variations in study participants’ characteristics, definitions of medication adherence, and sample sizes may account for the wide range of reported risk reductions. More importantly, the small number of studies from only 3 countries (Australia, China, and New Zealand) limits the generalizability of the findings to other settings. The methods used to assess adherence were different among the included studies, making it difficult to fully understand the effectiveness of the implemented interventions on adherence. This is particularly important given the absence of a gold-standard adherence measurement or adequate level of adherence that could predict health outcomes. In the literature, there are different types, measurement tools, and definitions of what constitutes medication adherence, bringing substantial heterogeneity between clinical studies [33,34]. Generating evidence from this meta-analysis may have been affected by these differences, depending on the amount to which these variations were present. Therefore, owing to the limited number of studies with strict inclusion criteria, variations in the ways interventions were delivered, and differences in health service delivery, the overall generalizability of our findings is limited. Finally, as only English-language papers were selected, it is possible that some important studies may have been missed during the literature search.

### Conclusions

The current meta-analysis revealed that there is limited evidence on the effectiveness of eHealth interventions on adherence to guideline-recommended medications after ACS compared to controls. Although prior studies demonstrate the use of eHealth tools for modifying cardiovascular risk factors, our pooled analysis of RCTs specific to people who had ACS did not find a significant association between eHealth interventions and medication adherence across 5 standard drug classes at 6- and 12-month follow-ups. This highlights the need for further studies to better understand the role of different eHealth approaches, including those beyond text messaging, to enhance post-ACS pharmacotherapy adherence and potentially resultant cardiovascular outcomes.

## Appendix

Multimedia Appendix 1 PRISMA checklist.

Multimedia Appendix 2 Search strategy.

Multimedia Appendix 3 Effect of eHealth intervention on medication adherence after acute coronary syndrome. ACEI: angiotensin-converting enzyme inhibitor; ARB: angiotensin receptor blocker [13-17].

Multimedia Appendix 4 Subgroup analysis: effect of eHealth intervention on medication adherence to cardioprotective therapies at 6 and 12-month.

## Abbreviations

ACEI: angiotensin-converting enzyme inhibitor
ACS: acute coronary syndrome
ARB: angiotensin receptor blocker
mHealth: mobile health
MMAS-8: 8-item Morisky medication adherence scale
PRISMA: Preferred Reporting Items for Systematic Reviews and Meta-Analysis
RCT: randomized controlled trial
RD: risk difference

## Data Availability

All data generated or analyzed during this study are included in this published article.
