# Supplementary material for: Efficacy of eHealth Technologies on Medication Adherence in Patients With Acute Coronary Syndrome: Systematic Review and Meta-Analysis
Source: JMIR Cardio. 2023 Dec 19;7:e52697. doi: 10.2196/52697 (PMC10762619; doi:10.2196/52697)
Supplement: Multimedia Appendix 2 [file cardio_v7i1e52697_app2.docx]

| **PubMed** | | **Search Date (22/10/23)** |
| --- | --- | --- |
| **#1** | ((telemedicine[MH] \| ehealth[tiab] \| e-health[tiab] \| electronic health[tiab] \| telemedicine[tiab] \| telehealth[tiab] \| mhealth[tiab] \| m-health[tiab] \| mobile health[tiab] \| emedicine[tiab] \| e-medicine[tiab] \| etherap*[tiab] \| health technolog*[tiab] \| information technolog*[tiab] \| communication technolog* [tiab] \| mobile tech*[tiab] \| platform[TiAb] \| telecare[tiab] \| tele-care [tiab] \|telepharmacy [tiab] \| telecommunic*[tiab] \| telemonitoring [tiab] \|remote monitor* [tiab] \| remote consult* [tiab]\| telephone[MH] \| phone[TiAB] \| smartphone*[tiab] \| mobile device*[tiab] \| personal digital assistant[tiab] \| pda[tiab] \| wearable*[tiab] \| smartwatch*[tiab] \| smart-watch*[tiab] \| computers[MH] \| computers[TiAb] \| computer[TiAb] \| internet[MH] \| internet[TiAb] \| web[TiAb] \| website [tiab] \| e-mail[TiAb] \| email[TiAb] \| electronic mail[TiAb] \|online[TiAb] \| wireless[TiAb] \| bluetooth[tiab] \| blue tooth[tiab] \| mobile applications[MH] \| apps[tiab] \|app[tiab]\| mobile application[tiab] \| interactive media[tiab] \| social media[tiab] \| instant messag*[tiab] \| IM[tiab] \| text messaging[MH] \| text messag*[tiab] \| SMS[tiab] \| multimedia[tiab] \| MMS[tiab] \| chat[tiab] \| social network[tiab] \| teleconference*[tiab] \| videoconference*[tiab] \| virtual[tiab] \| digital[TiAb] \| (tablet*[TiAb] AND (mac \| ipad \| android \| Microsoft \| windows))) \| "telemedicine" [mesh] OR telemedicine [tiab] OR telemonitoring [tiab] OR mhealth [tiab] OR ehealth[tiab] OR "m health" [tiab] OR "mobile health" [tiab] OR smartphone[tiab] OR "smart phone" [tiab] OR "smartphone" [mesh] OR "Cell phones" [tiab] OR "phone based" [tiab] OR "mobile based" [tiab] OR "phone app" [tiab] OR "mobile app" [tiab] OR "smartphone app" [tiab] OR "Mobile Applications" [Mesh] OR "Mobile Applications"[tiab]) AND (cell phone) | 24,781 |
| **#2** | ("medication adherence"[TIAB] OR "medication compliance"[TIAB] OR "medication nonadherence"[TIAB] OR "medication noncompliance"[TIAB] OR "medication non-adherence"[TIAB] OR "medication non-compliance"[TIAB] OR "drug adherence"[TIAB] OR "drug compliance"[TIAB] OR "drug nonadherence"[TIAB] OR "drug noncompliance"[TIAB] OR "drug non-compliance"[TIAB] OR "drug non-adherence"[TIAB] OR "medication persistence"[TIAB] OR "patient compliance"[TIAB] OR "patient adherence"[TIAB]) | 36,648 |
| **#3** | ("acute coronary syndrome"[MeSH] OR "acute coronary syndrome#"[tiab] OR "myocardial infarction"[MeSH] OR "myocardial infarction"[tiab] OR "ACS"[tiab] OR "coronary syndrome"[tiab] OR "acute myocardial ischemia"[tiab] OR "unstable angina"[tiab] OR "NSTEMI"[tiab] OR "STEMI"[tiab] OR "heart attack"[tiab] OR "coronary artery disease"[tiab] OR "cardiac ischemia"[tiab] OR "coronary thrombosis"[tiab]) | 305,644 |
| **#4** | #1 AND #2 AND #3 | 24 |
| **Embase** | | |
| **#1** | ((telemedicine/ or ehealth.tw. or e-health.tw. or electronic health.tw. or telemedicine.tw. or telehealth.tw. or mhealth.tw. or m-health.tw. or mobile health.tw. or emedicine.tw. or e-medicine.tw. or etherap*.tw. or health technolog*.tw. or information technolog*.tw. or communication technolog*.tw. or mobile tech*.tw. or platform.tw. or telecare.tw. or tele-care.tw. or telepharmacy.tw. or telecommunic*.tw. or telemonitoring.tw. or remote monitor*.tw. or remote consult*.tw. or telephone/ or phone.tw. or smartphone*.tw. or mobile device*.tw. or personal digital assistant.tw. or pda.tw. or wearable*.tw. or smartwatch*.tw. or smart-watch*.tw. or computers/ or computers.tw. or computer.tw. or internet/ or internet.tw. or web.tw. or website.tw. or e-mail.tw. or email.tw. or electronic mail.tw. or online.tw. or wireless.tw. or bluetooth.tw. or blue tooth.tw. or mobile applications/ or apps.tw. or app.tw. or mobile application.tw. or interactive media.tw. or social media.tw. or instant messag*.tw. or IM.tw. or text messaging/ or text messag*.tw. or SMS.tw. or multimedia.tw. or MMS.tw. or chat.tw. or social network.tw. or teleconference*.tw. or videoconference*.tw. or virtual.tw. or digital.tw. or (tablet*.tw. and (mac or ipad or android or Microsoft or windows))) or (telemedicine/ or telemonitoring.tw. or mhealth.tw. or ehealth.tw. or "m health".tw. or "mobile health".tw. or smartphone.tw. or "smart phone".tw. or "smartphone".tw. or "Cell phones".tw. or "phone based".tw. or "mobile based".tw. or "phone app".tw. or "mobile app".tw. or "smartphone app".tw. or "Mobile Applications"/ or "Mobile Applications".tw.) and (cell phone/ or mobile phone/ or cellular phone/)) | 18658 |
| **#2** | ("Medication adherence" OR "Medication compliance" OR "Medication nonadherence" OR "Medication noncompliance" OR "Medication non-adherence" OR "Medication non-compliance" OR "Drug adherence" OR "Drug compliance" OR "Drug nonadherence" OR "Drug noncompliance" OR "Drug non-adherence" OR "Drug non-compliance" OR "Medication persistence" OR "Patient compliance" OR "Patient adherence") | 207,725 |
| **#3** | 'acute coronary syndrome'/ or 'acute coronary syndrome#'.ti,ab. or 'myocardial infarction'/ or 'myocardial infarction#'.ti,ab. or 'ACS'.ti,ab. or 'coronary syndrome'.ti,ab. or 'acute myocardial ischemia'.ti,ab. or 'unstable angina'.ti,ab. or 'NSTEMI'.ti,ab. or 'STEMI'.ti,ab. or 'heart attack'.ti,ab. or 'coronary artery disease'.ti,ab. or 'cardiac ischemia'.ti,ab. or 'coronary thrombosis'.ti,ab. | 411,632 |
| **#4** | #1 AND #2 AND #3 | 9 |
| **Scopus** | | |
| **#1** | (TITLE-ABS-KEY(telemedicine) OR TITLE-ABS-KEY(ehealth) OR TITLE-ABS-KEY(e-health) OR TITLE-ABS-KEY("electronic health") OR TITLE-ABS-KEY(telehealth) OR TITLE-ABS-KEY(mhealth) OR TITLE-ABS-KEY(m-health) OR TITLE-ABS-KEY("mobile health") OR TITLE-ABS-KEY(emedicine) OR TITLE-ABS-KEY(e-medicine) OR TITLE-ABS-KEY(etherap*) OR TITLE-ABS-KEY("health technolog*") OR TITLE-ABS-KEY("information technolog*") OR TITLE-ABS-KEY("communication technolog*") OR TITLE-ABS-KEY("mobile tech*") OR TITLE-ABS-KEY(platform) OR TITLE-ABS-KEY(telecare) OR TITLE-ABS-KEY(tele-care) OR TITLE-ABS-KEY(telepharmacy) OR TITLE-ABS-KEY(telecommunic*) OR TITLE-ABS-KEY(telemonitoring) OR TITLE-ABS-KEY("remote monitor*") OR TITLE-ABS-KEY("remote consult*") OR TITLE-ABS-KEY(telephone) OR TITLE-ABS-KEY(phone) OR TITLE-ABS-KEY("smartphone*") OR TITLE-ABS-KEY("mobile device*") OR TITLE-ABS-KEY("personal digital assistant") OR TITLE-ABS-KEY(pda) OR TITLE-ABS-KEY(wearable*) OR TITLE-ABS-KEY("smartwatch*") OR TITLE-ABS-KEY("smart-watch*") OR TITLE-ABS-KEY(computers) OR TITLE-ABS-KEY(computer) OR TITLE-ABS-KEY(internet) OR TITLE-ABS-KEY(web) OR TITLE-ABS-KEY(website) OR TITLE-ABS-KEY("e-mail") OR TITLE-ABS-KEY(email) OR TITLE-ABS-KEY("electronic mail") OR TITLE-ABS-KEY(online) OR TITLE-ABS-KEY(wireless) OR TITLE-ABS-KEY(blue*tooth) OR TITLE-ABS-KEY("mobile applications") OR TITLE-ABS-KEY(apps) OR TITLE-ABS-KEY(app) OR TITLE-ABS-KEY("mobile application") OR TITLE-ABS-KEY("interactive media") OR TITLE-ABS-KEY("social media") OR TITLE-ABS-KEY("instant messag*") OR TITLE-ABS-KEY(IM) OR TITLE-ABS-KEY("text messaging") OR TITLE-ABS-KEY("text messag*") OR TITLE-ABS-KEY(SMS) OR TITLE-ABS-KEY(multimedia) OR TITLE-ABS-KEY(MMS) OR TITLE-ABS-KEY(chat) OR TITLE-ABS-KEY("social network") OR TITLE-ABS-KEY("teleconference*") OR TITLE-ABS-KEY("videoconference*") OR TITLE-ABS-KEY(virtual) OR TITLE-ABS-KEY(digital) OR TITLE-ABS-KEY(tablet* AND (mac OR ipad OR android OR Microsoft OR windows))) | 10,880,720 |
| **#2** | (TITLE-ABS-KEY("Medication adherence") OR TITLE-ABS-KEY("Medication compliance") OR TITLE-ABS-KEY("Medication nonadherence") OR TITLE-ABS-KEY("Medication noncompliance") OR TITLE-ABS-KEY("Medication non-adherence") OR TITLE-ABS-KEY("Medication non-compliance") OR TITLE-ABS-KEY("Drug adherence") OR TITLE-ABS-KEY("Drug compliance") OR TITLE-ABS-KEY("Drug nonadherence") OR TITLE-ABS-KEY("Drug noncompliance") OR TITLE-ABS-KEY("Drug non-adherence") OR TITLE-ABS-KEY("Drug non-compliance") OR TITLE-ABS-KEY("Medication persistence") OR TITLE-ABS-KEY("Patient compliance") OR TITLE-ABS-KEY("Patient adherence")) | 211,326 |
| **#3** | ("acute coronary syndrome" OR "ACS" OR "coronary syndrome" OR "acute myocardial ischemia" OR "unstable angina" OR "NSTEMI" OR "STEMI" OR "heart attack" OR "coronary artery disease" OR "cardiac ischemia" OR "coronary thrombosis") | 7,066,682 |
| **#4** | (INDEXTERMS ( "clinical trial*" OR "randomized controlled trial" OR "controlled clinical trial" OR "Controlled Clinical Trials" OR "clinical trial*" OR "controlled study" OR "randomised controlled trial" ) ) | 8,761,965 |
| **#5** | #1 AND #2 AND #3 AND #4 | 909 |
| **Web of Science** | | |
| **#1** | TS=("telemedicine" OR "ehealth" OR "e-health" OR "electronic health" OR "telehealth" OR "mhealth" OR "m-health" OR "mobile health" OR "emedicine" OR "e-medicine" OR "etherap*" OR "health technology" OR "information technology" OR "communication technology" OR "mobile technology" OR "platform" OR "telecare" OR "tele-care" OR "telepharmacy" OR "telecommunication" OR "telemonitoring" OR "remote monitor" OR "remote consult" OR "telephone" OR "phone" OR "smartphone" OR "mobile device" OR "personal digital assistant" OR "pda" OR "wearable" OR "smartwatch" OR "smart-watch" OR "computer" OR "internet" OR "web" OR "website" OR "e-mail" OR "email" OR "electronic mail" OR "online" OR "wireless" OR "bluetooth" OR "mobile applications" OR "apps" OR "app" OR "mobile application" OR "interactive media" OR "social media" OR "instant message" OR "IM" OR "text messaging" OR "text message" OR "SMS" OR "multimedia" OR "MMS" OR "chat" OR "social network" OR "teleconference" OR "videoconference" OR "virtual" OR "digital" OR "tablet" OR ("mac" OR "ipad" OR "android" OR "Microsoft" OR "windows")) | 4,925,616 |
| **#2** | TS=(("medication adherence" OR "medication compliance" OR "medication nonadherence" OR "medication noncompliance" OR "medication non-adherence" OR "medication non-compliance" OR "drug adherence" OR "drug compliance" OR "drug nonadherence" OR "drug noncompliance" OR "drug non-adherence" OR "medication persistence" OR "patient compliance" OR "patient adherence")) | 47,033 |
| **#3** | TS=("acute coronary syndrome" OR "ACS" OR "coronary syndrome" OR "acute myocardial ischemia" OR "unstable angina" OR "NSTEMI" OR "STEMI" OR "heart attack" OR "coronary artery disease" OR "cardiac ischemia" OR "coronary thrombosis") | 250,328 |
| **#4** | TS=("clinical trial*" OR "randomized controlled trial" OR "controlled clinical trial" OR "Controlled Clinical Trials" OR "controlled study" OR "randomised controlled trial") | 814,025 |
| **#4** | #1 AND #2 AND #3 AND #4 | 62 |
