# Supplementary figures and images for: Efficacy of eHealth Technologies on Medication Adherence in Patients With Acute Coronary Syndrome: Systematic Review and Meta-Analysis
Source: JMIR Cardio. 2023 Dec 19;7:e52697. doi: 10.2196/52697 (PMC10762619; doi:10.2196/52697)

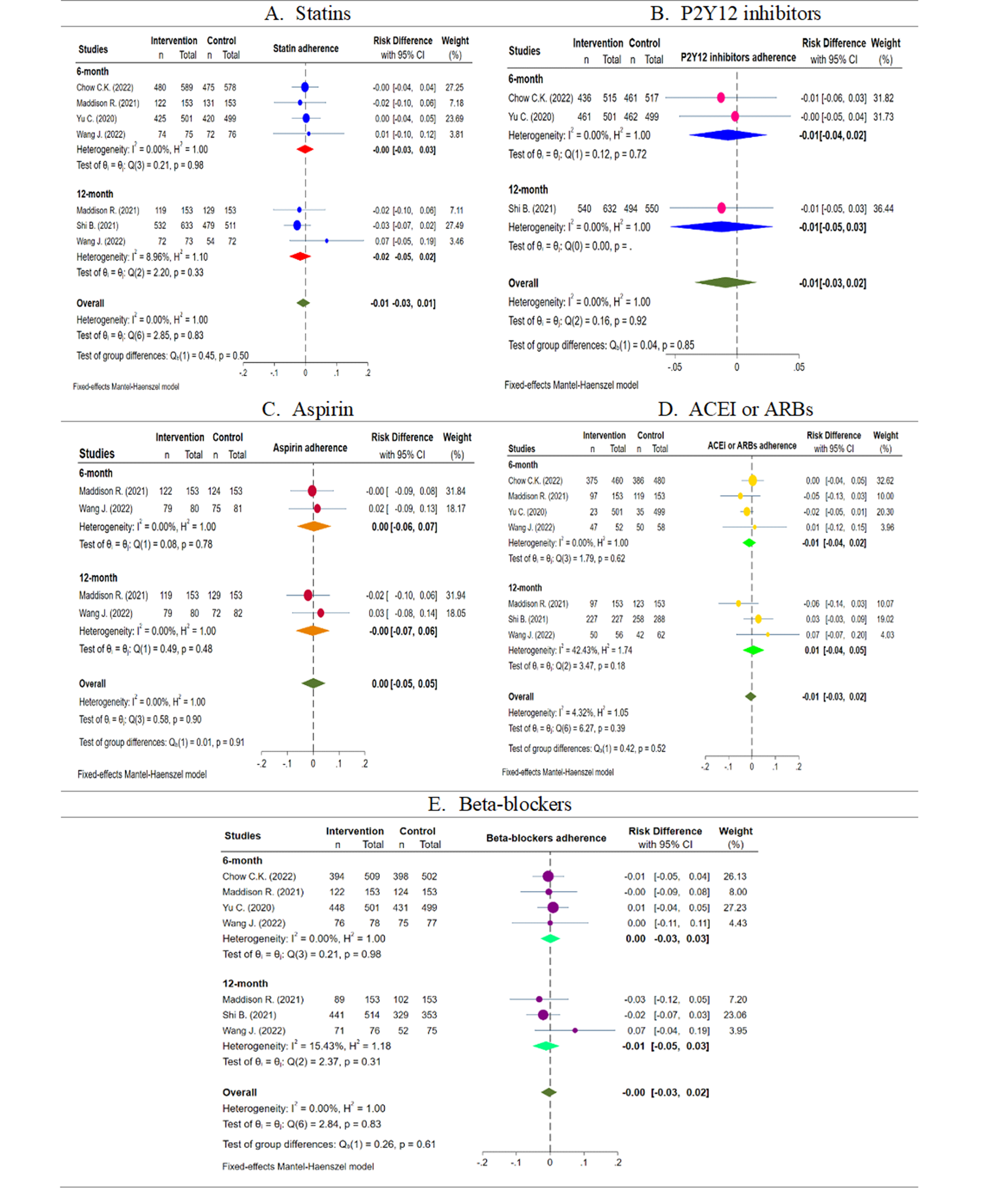

Supplement: Multimedia Appendix 3 [file cardio_v7i1e52697_app3.png]
