# Supplementary material for: Efficacy of eHealth Technologies on Medication Adherence in Patients With Acute Coronary Syndrome: Systematic Review and Meta-Analysis
Source: JMIR Cardio. 2023 Dec 19;7:e52697. doi: 10.2196/52697 (PMC10762619; doi:10.2196/52697)
Supplement: Multimedia Appendix 4 [file cardio_v7i1e52697_app4.docx]

| Adherence at 6-month | Adherence at 12-month |
| --- | --- |
| 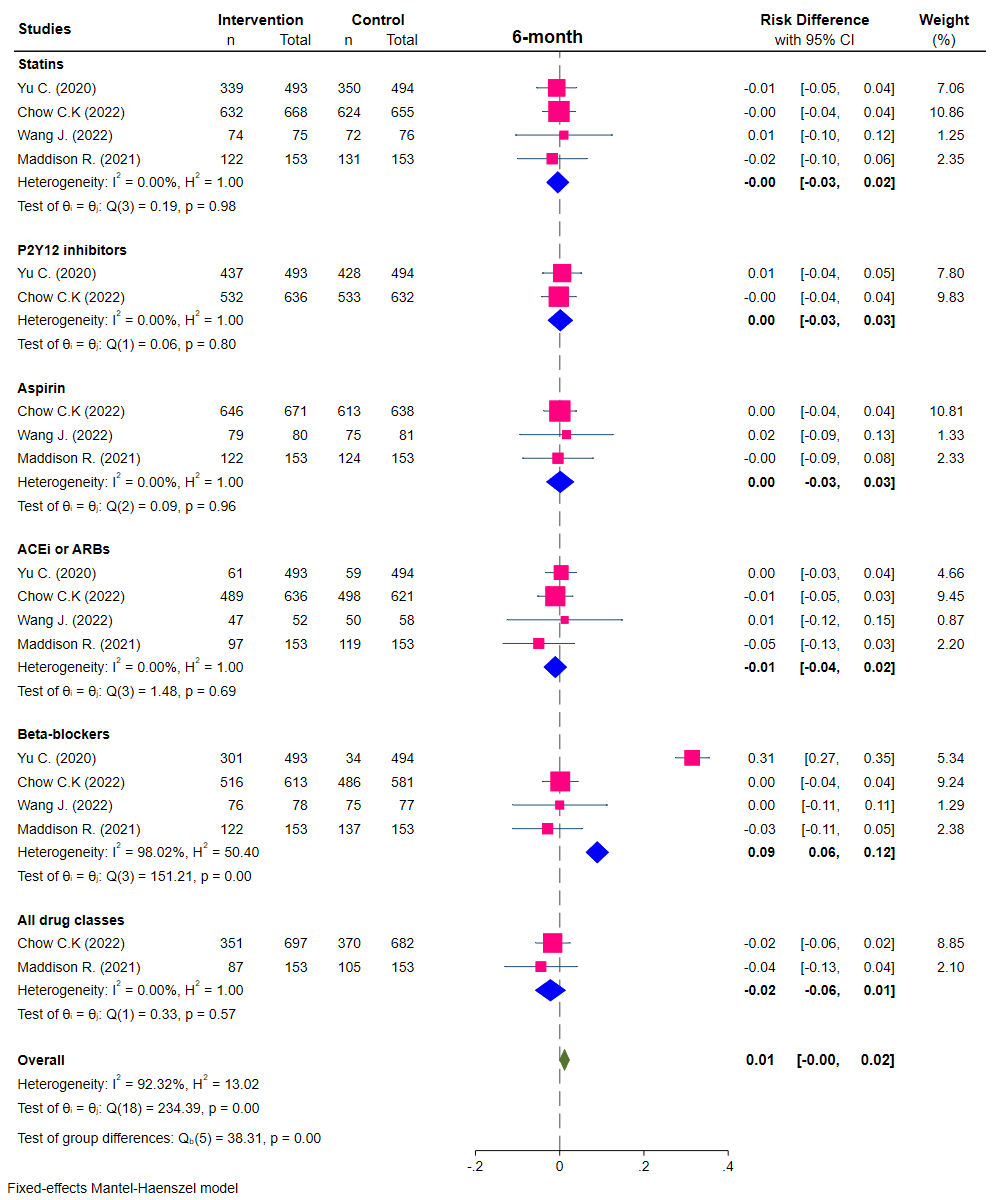 | 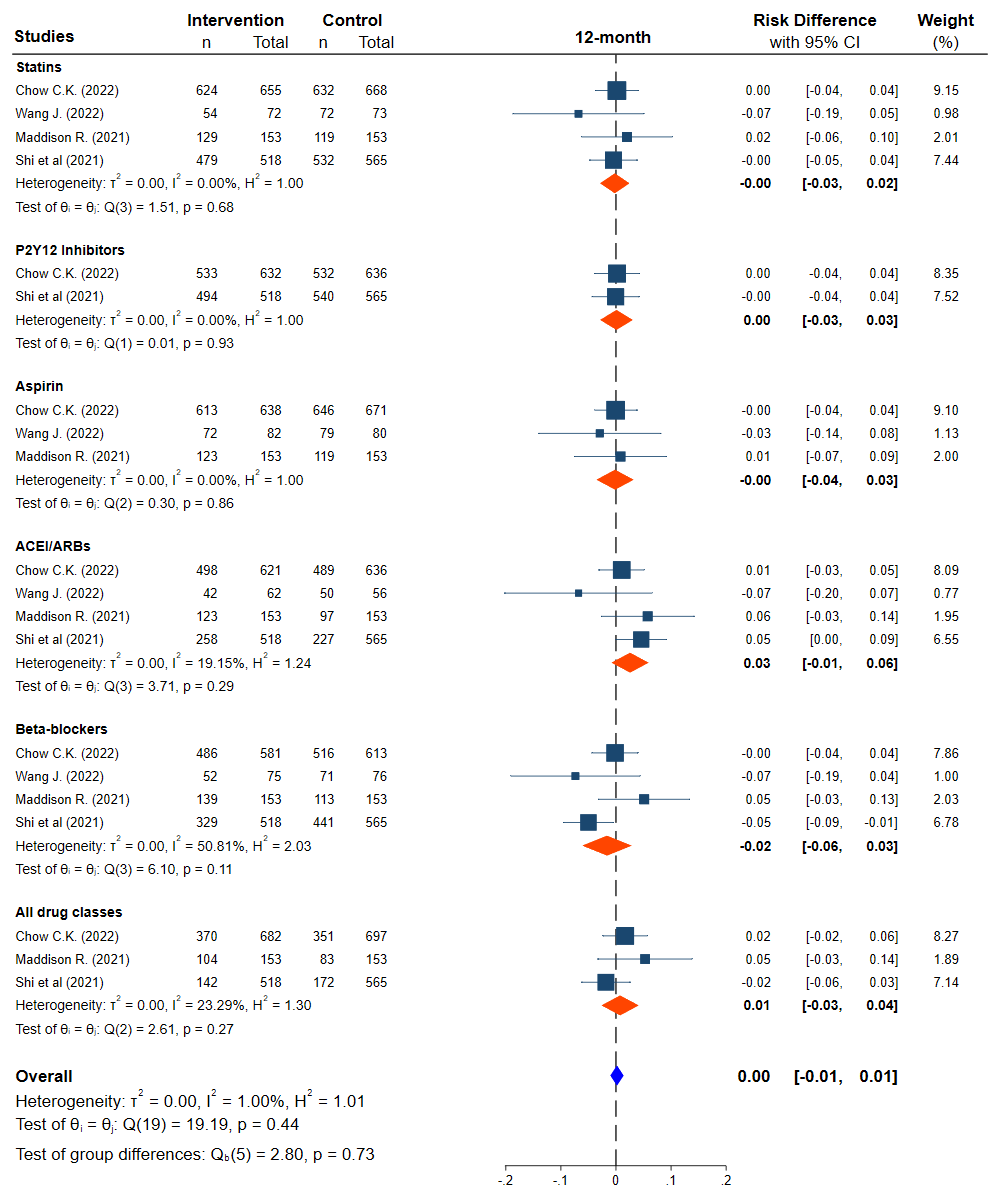 |

| Self-reported Adherence at 6-month | Self-reported Adherence at 12-month |
| --- | --- |
| 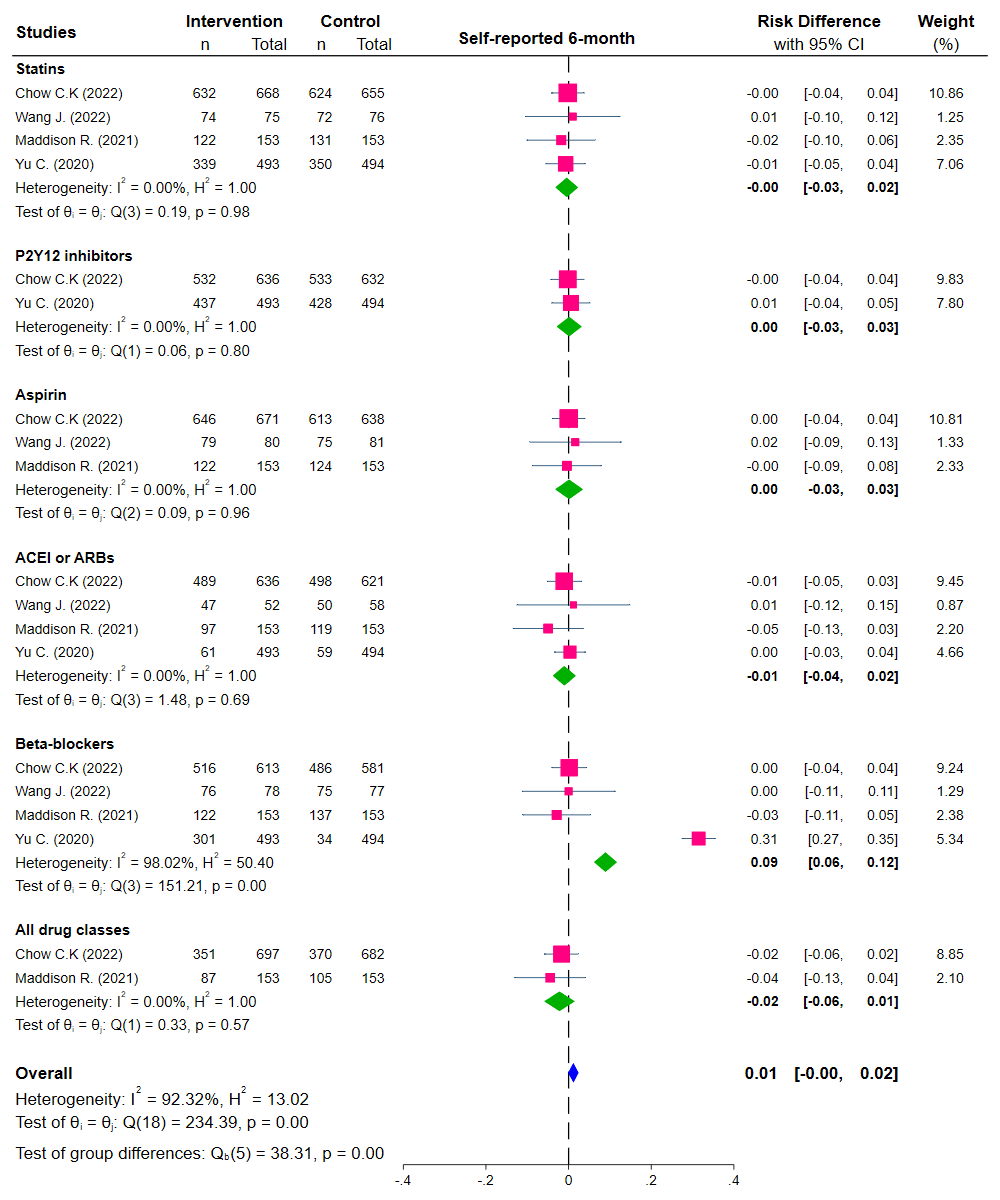 | 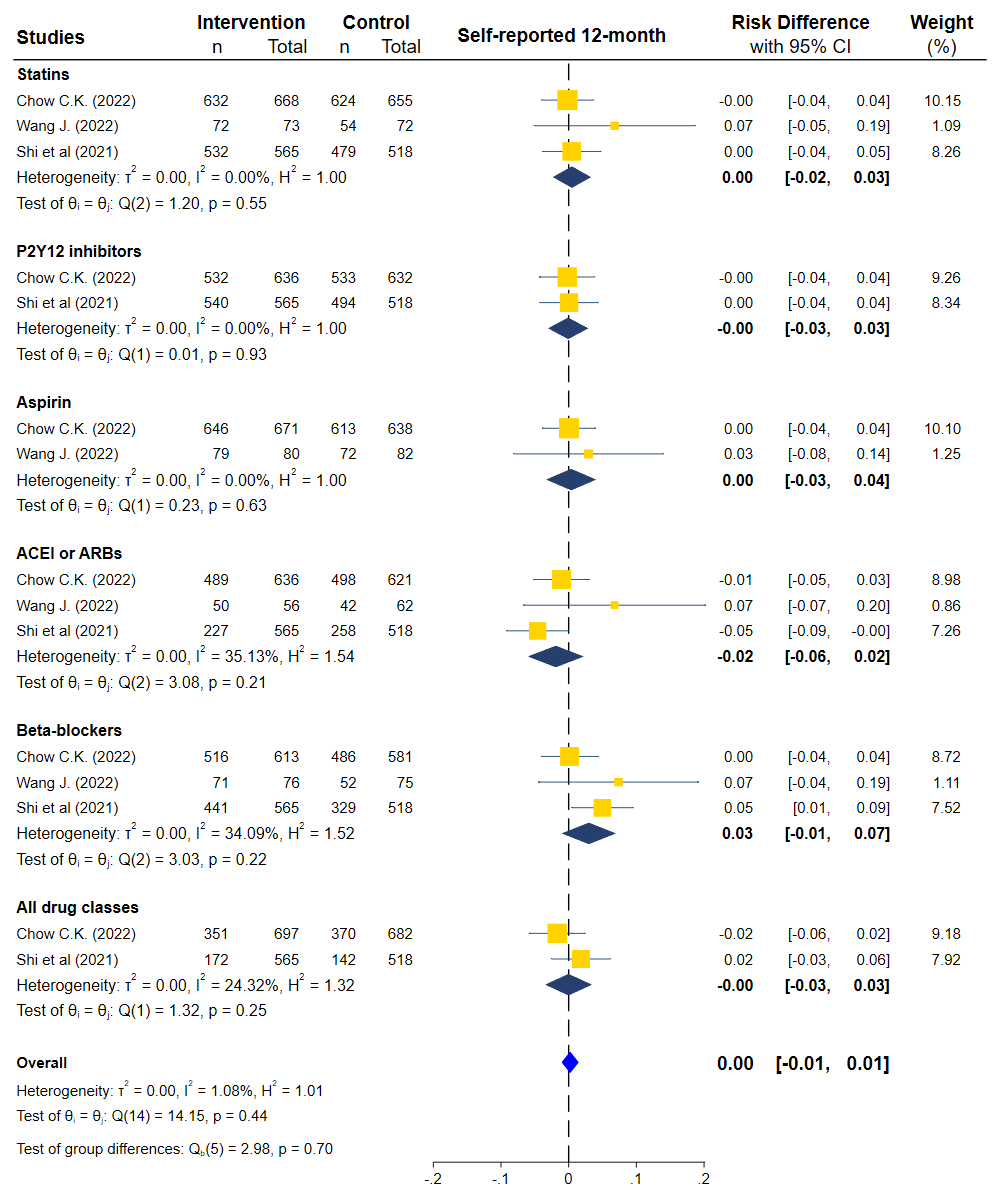 |

| 6-month objective assessment | 12-month objective assessment |
| --- | --- |
| 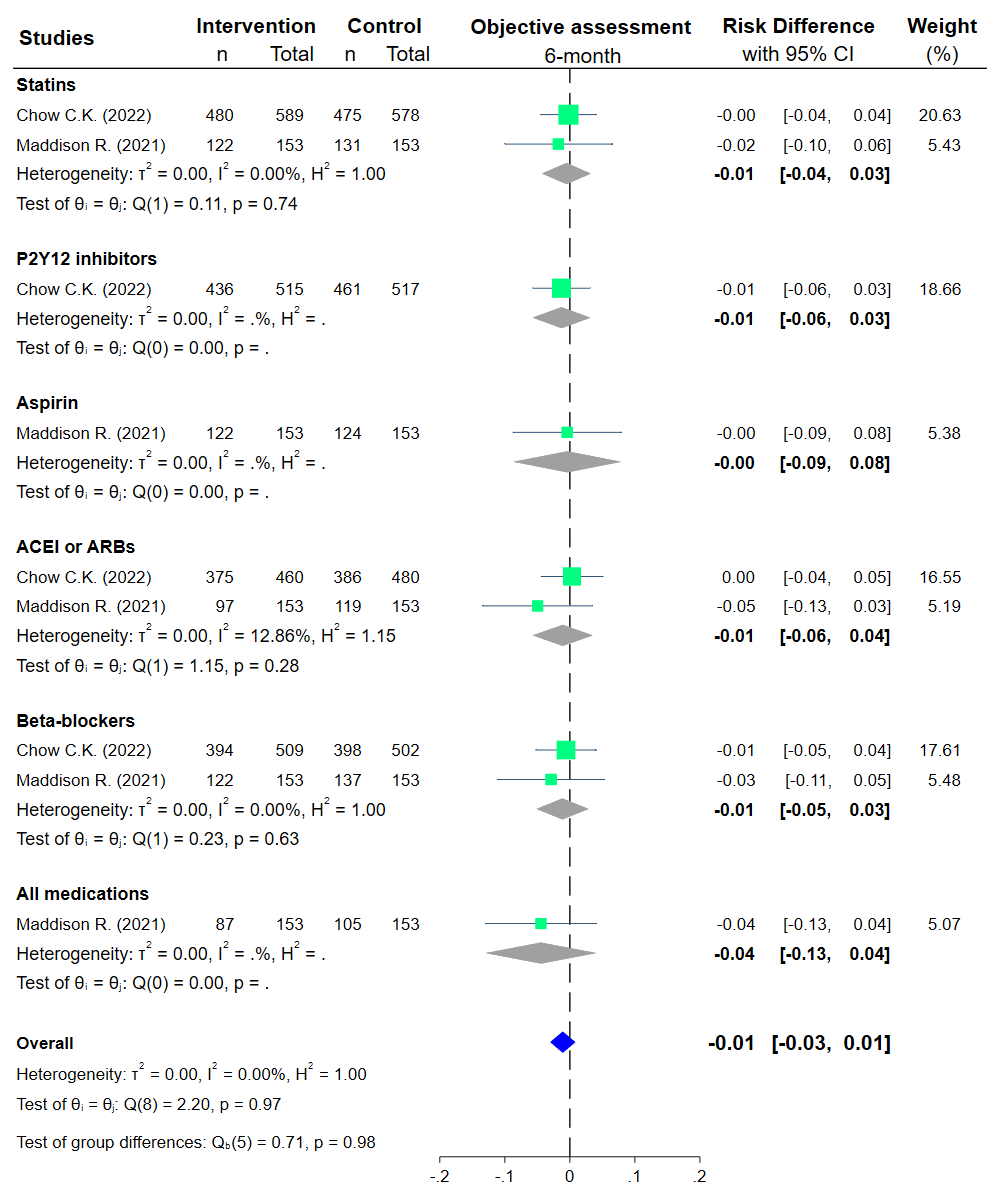 | 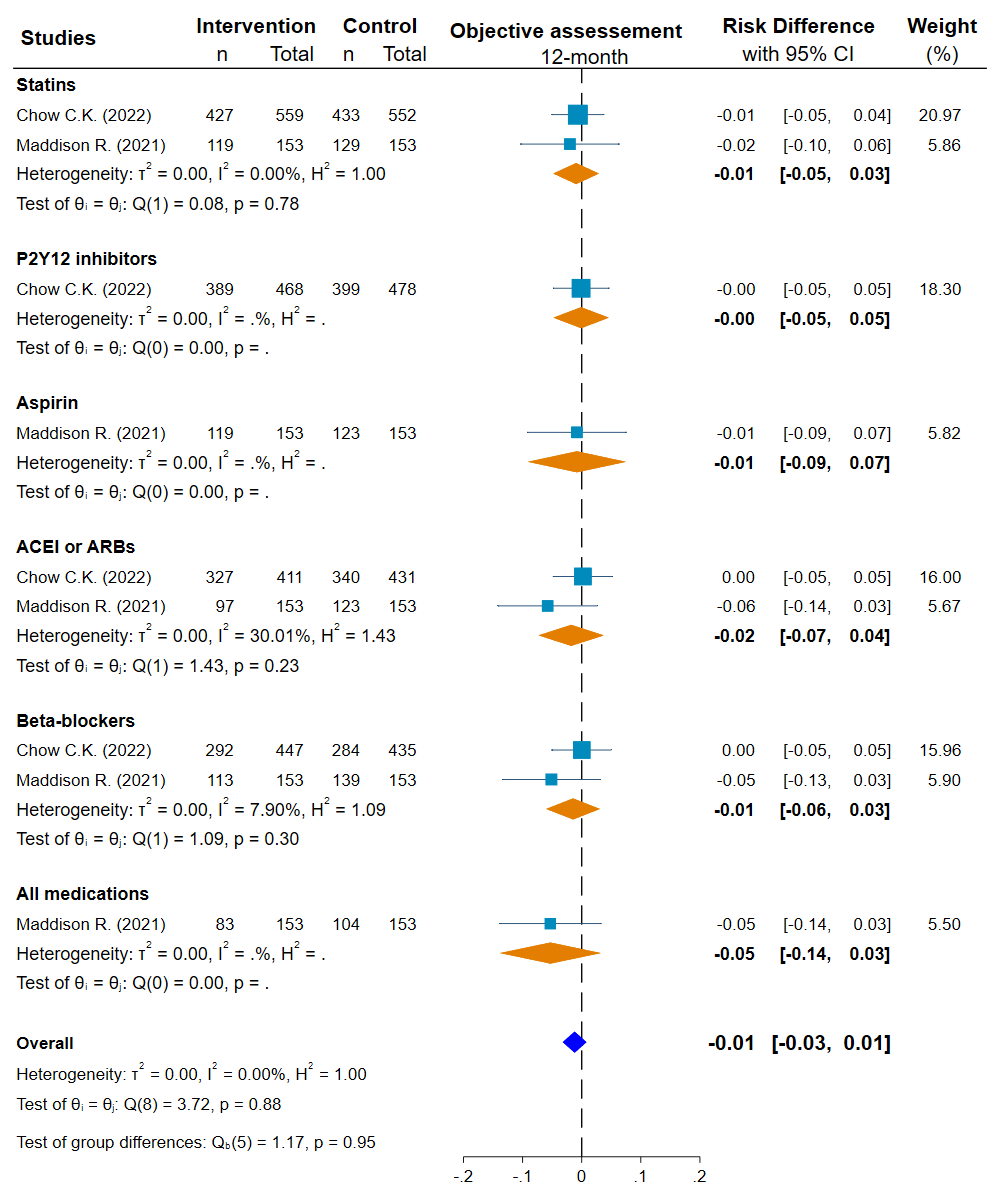 |
